# Capacidade Preditiva dos Parâmetros do Teste de Esforço Cardiopulmonar em Pacientes com Insuficiência Cardíaca em Terapia de Ressincronização Cardíaca

**DOI:** 10.36660/abc.20210620

**Published:** 2022-07-13

**Authors:** João Ferreira Reis, António Valentim Gonçalves, Pedro Garcia Brás, Rita Ilhão Moreira, Pedro Rio, Ana Teresa Timóteo, Rui M. Soares, Rui Cruz Ferreira

**Affiliations:** 1 Departamento de Cardiologia Hospital de Santa Marta Centro Hospitalar Central de Lisboa Lisboa Portugal Departamento de Cardiologia, Hospital de Santa Marta, Centro Hospitalar Central de Lisboa, Lisboa – Portugal

**Keywords:** Insuficiência Cardíaca, Terapia de Ressincronização Cardíaca/métodos, Teste de Esforço/métodos, Consumo de Oxigênio, Transplante do Coração

## Abstract

**Fundamento:**

Há evidências sugerindo que um corte do pico de consumo de oxigênio (pVO_2_) de 10ml/kg/min fornece uma estratificação de risco mais precisa em pacientes com Terapia de Ressincronização Cardíaca (TRC).

**Objetivo:**

Comparar o poder prognóstico de vários parâmetros do teste cardiopulmonar de exercício (TCPE) nesta população e avaliar a capacidade discriminativa dos valores de corte de pVO_2_ recomendados pelas diretrizes.

**Métodos:**

Avaliação prospectiva de uma série consecutiva de pacientes com insuficiência cardíaca (IC) com fração de ejeção do ventrículo esquerdo ≤40%. O desfecho primário foi um composto de morte cardíaca e transplante cardíaco urgente (TC) nos primeiros 24 meses de acompanhamento, e foi analisado por vários parâmetros do TCPE para a maior área sob a curva (AUC) no grupo TRC. Uma análise de sobrevida foi realizada para avaliar a estratificação de risco fornecida por vários pontos de corte diferentes. Valores de p < 0,05 foram considerados significativos.

**Resultados:**

Um total de 450 pacientes com IC, dos quais 114 possuíam aparelho de TRC. Esses pacientes apresentaram um perfil de risco basal mais alto, mas não houve diferença em relação ao desfecho primário (13,2% vs 11,6%, p = 0,660). A pressão expiratória de dióxido de carbono no limiar anaeróbico (P_ET_CO_2AT_) teve o maior valor de AUC, que foi significativamente maior do que o de pVO_2_ no grupo TRC (0,951 vs 0,778, p = 0,046). O valor de corte de pVO2 atualmente recomendado forneceu uma estratificação de risco precisa nesse cenário (p <0,001), e o valor de corte sugerido de 10 ml/min/kg não melhorou a discriminação de risco em pacientes com dispositivos (p = 0,772).

**Conclusão:**

A P_ET_CO_2AT_ pode superar o poder prognóstico do pVO_2_ para eventos adversos em pacientes com TRC. O ponto de corte de pVO_2_ recomendado pelas diretrizes atuais pode estratificar precisamente o risco dessa população.

## Introdução

O teste cardiopulmonar de exercício (TCPE) é um poderoso preditor de mortalidade em pacientes com insuficiência cardíaca com fração de ejeção reduzida (ICFER) e é utilizado para orientar o encaminhamento de pacientes para terapias avançadas, como transplante cardíaco (TC) e suporte circulatório mecânico (MCS).^1 - 3^

O pico de consumo de oxigênio (pVO_2_) e o slope VE/VCO2 são as variáveis derivadas do TCPE mais comumente usadas como ferramentas de avaliação de risco, no entanto, várias outras variáveis do TCPE demonstraram predizer eventos de IC e, algumas delas, podem melhorar a estratificação clínica da pacientes com IC quando usadas em conjunto com as variáveis acima mencionadas (ou seja, ventilação oscilatória de exercício, variação de dióxido de carbono expirado durante o teste de esforço, recuperação da FC, pressão arterial sistólica e resposta do ECG ao exercício).

A terapia de ressincronização cardíaca (TRC) surgiu como uma importante opção terapêutica no manejo de pacientes com ICFER e, em pacientes selecionados, demonstrou melhorar a carga sintomática e a qualidade de vida, além de ter um benefício prognóstico em relação à morbidade e mortalidade.^4 - 8^ Um número crescente de pacientes encaminhados a TC já possui um aparelho de TRC, com ou sem desfibrilador (TRC-D e TRC-P, respectivamente). A sobrevida em pacientes com ICFER melhorou significativamente nos últimos anos e alguns autores sugerem a necessidade de reavaliação dos critérios de listagem para TC e limiares prognósticos de pico de consumo de oxigênio (pVO_2_) e slope VE/VCO^2^ .^9 , 10^

Os critérios de listagem para transplante cardíaco da *International Society for Heart Lung Transplantation* (ISHLT) de 2016 definiram o pVO_2_ como critério principal para listar pacientes para TC e que a presença do dispositivo TRC não altera o valor de corte recomendado de pVO_2._^11^ Essa recomendação foi baseada em uma subanálise do estudo COMPANION que mostrou que a TRC não alterou a previsibilidade do pVO_2_ em eventos adversos de ICFER.^12 , 13^ Por outro lado, Goda et al.,^14^ mostraram que um valor de corte de 10 ml/kg/min em vez do valor de corte tradicional de 14 ml/kg/min pode ser mais útil para estratificação de risco em pacientes com TRC.^14^ Várias outras variáveis do TCPE demonstraram ser preditores robustos de pior desfecho clínico em populações com ICFER, como o slope VE/VCO2, o slope da eficiência de captação de O2 (OUES) e o ponto ótimo cardiorrespiratório (COP).^15 , 16^

O presente estudo busca avaliar a capacidade preditiva dos valores de corte recomendados pelas diretrizes em pacientes com TRC, comparar o poder prognóstico de vários parâmetros de exercício com o do pVO_2_ nesta população e comparar seu desempenho entre pacientes com e sem dispositivo de TRC .

## Métodos

### Considerações éticas

Essa pesquisa está em conformidade com os princípios descritos na Declaração de Helsinque. O comitê de ética local aprovou o protocolo do estudo. Todos os pacientes forneceram consentimento informado.

### Amostra de estudo

Análise de um único centro de uma série consecutiva de 450 pacientes com IC encaminhados à nossa instituição de 2009 a 2018 com fração de ejeção do ventrículo esquerdo (FEVE) ≤40% e classe II ou III da *New York Heart Association* (NYHA), que foram submetidos a TCPE. Todos os pacientes foram encaminhados para avaliação pela equipe de HF com possível indicação para TC ou MCS.

### Protocolo de estudo

O acompanhamento dos pacientes incluiu avaliação inicial no período de um mês em cada paciente com: dados clínicos incluindo etiologia da IC (isquêmica vs não isquêmica), dispositivos cardíacos implantados (CIED), medicação, comorbidades, classe NYHA; dados laboratoriais; dados eletrocardiográficos; dados ecocardiográficos; dados TCPE; Escore de Sobrevida à Insuficiência Cardíaca (HFSS).

Os pacientes foram excluídos por algum dos seguintes critérios: idade <18 anos; revascularização coronária percutânea planejada ou cirurgia cardíaca; comorbidades limitantes ao exercício (doença cerebrovascular, comprometimento musculoesquelético ou doença vascular periférica grave); TC anterior.

Os pacientes submetidos ao implante de TRC realizaram TCPE e ecocardiograma transtorácico pelo menos 6 meses após o procedimento.

Pacientes com TC eletivo durante o período de seguimento (pacientes que tiveram indicação de TC e coração foi disponibilizado nos primeiros dois anos de seguimento) foram censurados da análise no momento do TC.

### Teste de esforço cardiopulmonar

Um TCPE em esteira com sintomas máximos limitados, definido pelo pico da taxa de troca respiratória (RER) > 1,05, foi realizada usando o protocolo de Bruce modificado (esteira GE Marquette Series 2000). A análise dos gases foi precedida pela calibração do equipamento. Ventilação por minuto, consumo de oxigênio e produção de dióxido de carbono foram adquiridos respiração a respiração, usando um analisador de gases SensorMedics Vmax 229.

O pVO_2_ foi definido como a maior média de 30 segundos alcançada durante o exercício e foi normalizado para a massa corporal. O limiar anaeróbio foi determinado pela combinação dos métodos padrão (preferencialmente V-slope e equivalentes ventilatórios). O slope VE/VCO2 foi calculado por regressão linear de mínimos quadrados, utilizando dados adquiridos ao longo de todo o exercício. O COP foi medido como o valor mínimo do equivalente ventilatório para oxigênio (VE/VO2 mínimo). A pressão parcial de dióxido de carbono expirado (P_ET_CO_2_) foi relatada antes do exercício (P_ET_CO_2AR_), no limiar anaeróbio (P_ET_CO_2AT_) e no pico do exercício em unidades de mmHg, e também foi calculad o aumento durante o exercício até o limiar anaeróbio ser atingido (P_ET_CO_2DIF_). O pico de pulso de oxigênio (PP) foi calculado dividindo-se o pVO_2_ derivado pela frequência cardíaca (FC) máxima durante o exercício e foi expresso em mililitros por batimento. A potência circulatória foi calculada como o produto da pVO_2_ e o pico da pressão arterial sistólica e a potência ventilatória foi calculada pela divisão do pico da pressão arterial sistólica (PA) pelo slope VE/VCO2. Vários parâmetros compostos do TCPE também foram calculados automaticamente.

### Acompanhamento e desfecho

Todos os pacientes foram acompanhados por 24 meses a partir da data de realização dos exames complementares supracitados.

O desfecho primário foi um composto de morte cardíaca e TC urgente ocorrendo durante uma hospitalização não planejada com dependência de inotrópicos para piora da IC. Os dados foram obtidos a partir das consultas ambulatoriais (ou seja, visitas não planejadas para IC – deterioração clínica com necessidade de diuréticos iv – ou visitas planejadas para titulação de medicamentos para IC, terapia diurética ou avaliação clínica de rotina pela equipe de IC) e foram complementados com um questionário padronizado de entrevista por telefone a todos os pacientes aos 24 meses de seguimento.

### Análise estatística

Todas as análises comparam pacientes com e sem dispositivo TRC (TRC e sem TRC, respectivamente). Os dados foram analisados por meio do software Statistical Package for the Social Science for Windows, versão 24.0 (SPSS Inc, Chicago IL).

As características basais foram resumidas em frequências (porcentagens) para variáveis categóricas, como médias e desvios-padrão para variáveis contínuas quando a normalidade foi verificada e como mediana e intervalo interquartil quando a normalidade não foi verificada pelo teste de Kolmogorov-Smirnov. O teste t de Student para amostras independentes ou o teste de Mann-Whitney (quando a normalidade não foi confirmada) foram utilizados para todas as comparações. O teste Qui-Quadrado ou o teste exato de Fisher foram usados para comparar as variáveis categóricas.

A análise multivariada para a predição do desfecho primário durante dois anos de seguimento foi realizada por meio da regressão de Cox, incluindo todas as variáveis estatisticamente significativas na análise univariada, na coorte total e em cada grupo.

O poder preditivo de vários parâmetros do TCPE em relação ao desfecho primário em cada grupo foi analisado com a curva Receiver Operating Characteristics (ROC) e área sob a curva (AUC). Os valores de corte para as variáveis foram determinados a partir das curvas ROC para que a soma da sensibilidade e especificidade fosse maximizada. O teste de Hanley e McNeil foi utilizado para comparar duas curvas ROC correlacionadas.^17^

A sobrevida livre de eventos foi determinada usando o método de Kaplan-Meier e comparada com a análise de log-rank para avaliar a capacidade discriminativa de risco fornecida pelos valores de corte de pVO_2_ recomendados pelas diretrizes (pVO_2_≤ 12 ml/kg/min ou ≤ 14 ml/kg/min sem betabloqueador - BB) e slope VE/VCO_2_^11^ e o valor de corte sugerido de 10ml/kg/min.^14^ Diferenças estatísticas com valor de p < 0,05 foram consideradas significativas.

## Resultados

### Visão geral dos grupos TRC e sem TRC

Um total de 450 pacientes foram incluídos no estudo, dos quais 25,3% (n = 114) tinham um dispositivo TRC, principalmente um TRC-D (98,2%). A população geral apresentou média de idade de 56,2 anos, sendo 78,7% do sexo masculino e FEVE média de 28,6%. Todos os pacientes com TRC com fibrilação atrial foram submetidos à ablação do nó AV durante o procedimento de implantação e a porcentagem de estimulação biventricular foi de 96%. O TCPE foi realizado em média 8 meses após o implante do TRC. As características basais de ambos os grupos são apresentadas na Tabela 1 .

**Tabela 1:** Características de linha de base dos grupos TRC e sem TRC

			Geral n 450	TRC n 114	Sem TRC n 336	valor p
			DADOS CLÍNICOS – CARACTERÍSTICAS			
Idade			56,2 ± 12,5	62,3 ± 11,5	54,2 ± 12,2	< 0,001
Masculino (%)			354 (78,7%)	85 (74,6%)	269 (80,1%)	0,216
IMC(kg/m^2^)			27,2 ± 4,3	27,2 ± 4,1	27,1 ± 4,4	0,829
Etiologia isquêmica (%)			211 (46,9%)	42 (36,8%)	169 (50,6%)	0,011
IECA/BRA/INRA (%)			423 (94,0%)	104 (96,3%)	319 (96,1%)	1,000
BB (%)			388 (86,2%)	93 (85,3%)	295 (88,9%)	0,325
MRA (%)			340 (75,6%)	93 (84,5%)	247 (74,2%)	0,026
Diabetes (%)			98 (21,8%)	23 (22,3%)	75 (23,4%)	0,817
**DRC (%)**			140 (31,1%)	48 (46,6%)	92 (32,1%)	**0,008**
**FA (%)**			112 (24,9%)	43 (38,1%)	69 (20,6%)	**< 0,001**
**CDI (%)**			271 (60,2%)	112 (98,2%)	159 (47,3%)	**< 0,001**
**Classe funcional NYHA**			2,2 ± 0,6	2,5 ± 0,5	2,1 ± 0,6	**0,001**
HFSS*			8,5 ± 1,0	8,14 ± 0,86	8,65 ± 1,04	**< 0,001**
			**DADOS LABORATORIAIS**			
**Creatinina (mg/dL)**			1,4 ± 0,7	1,6 ± 0,4	1,0 ± 0,3	**0,041**
Sódio (mEq/L)			137,9 ± 3,1	137,5 ± 3,4	138,5 ± 2,9	0,138
**NT-proBNP (pg/ml)**			2224,2 ± 2764,0	2769,7 ± 2575,4	2034,3 ± 2808,1	**0,045**
			**DADOS ECOCARDIOGRÁFICOS**			
**DDFVE (mm/m** ^**2**^ )*			35,5 ± 5,9	37,9 ± 5,5	34,7 ± 5,9	**0,032**
**FEVE (%)**			28,6 ± 6,9	26,2 ± 7,2	29,6 ± 6,6	**< 0,001**
MR III-IV (%)			65 (14,7%)	16 (14,0%)	49 (14,5%)	0,935
			**DADOS TCPE**			
**Duração TCPE (min)**			9,6 ± 4,4	7,4 ± 4,1	10,3 ± 4,3	**< 0,001**
Pico RER			1,07 ± 0,11	1,05 ± 0,11	1,08 ± 0,10	0,139
**pVO**_**2**_ **(ml/kg/min)**			17,9 ± 6,1	15,2 ± 5,1	18,8 ± 6,1	**< 0,001**
**Slope VE/VCO**_**2**_			33,8 ± 9,5	35,8 ± 10,9	33,2 ± 8,9	**0,026**
OUES			2,1 ± 1,8	2,2 ± 2,2	2,0 ± 1,6	0,645
**pVO**_**2**_ **(ml/kg/min) em AT**			13,1 ± 4,5	10,3 ± 3,4	13,8 ± 4,5	**0,001**
**Pulso de O**_**2**_ **(mL/kg/batimento)**			0,14 ± 0,06	0,12 ± 0,04	0,14 ± 0,07	**0,028**
**Potência Circulatória (mmHg.ml.kg-1 min-1)**			2786,9 ± 1578,8	2262,3 ± 965,4	2963 ± 1702,4	**< 0,001**
**Potência ventilatória (mmHg)**			4,8 ± 1,7	4,4 ± 1,7	4,9 ± 1,7	**0,020**
Ponto ótimo cardiorrespiratório			29,6 ± 7,4	30,7 ± 7,5	29,3 ± 7,4	0,274
P_ET_CO_2_ em repouso (mmHg)			33,4 ± 4,7	32,9 ± 4,8	33,6 ± 4,7	0,241
**P**_**ET**_ **CO**_**2AT**_ **(mmHg)**			36,7 ± 5,9	35,3 ± 5,9	37,1 ± 5,9	**0,010**
**P**_**ET**_ **CO**_**2DIF**_ **(mmHg)**			3,3 ± 3,7	2,3 ± 3,2	3,6 ± 3,8	**0,004**

Os valores são média ± desvio padrão ou mediana (intervalo interquartil); os valores p são calculados pelo teste T de Student para amostras independentes ou teste U de Mann-Whitney conforme apropriado; o teste do qui-quadrado ou o teste exato de Fisher foram usados para comparar as variáveis categóricas. *Variáveis com distribuição normal. AT: limiar anaeróbio; IECA: inibidores da enzima conversora de angiotensina; BRA: bloqueadores do receptor de angiotensina; INRA: inibidores do receptor de angiotensina-neprilisina; FA: Fibrilação atrial; BB: Betabloqueadores; IMC: índice de massa corporal; TCPE: teste cardiopulmonar de exercício; DRC: doença renal crônica; HFSS: Escore de Sobrevida à Insuficiência Cardíaca; CDI: cardioversor desfibrilador implantável; FEVE: fração de ejeção do ventrículo esquerdo; DDFVE: diâmetro diastólico final do ventrículo esquerdo; MRA: antagonistas do receptor mineralocorticóide; MR: Insuficiência mitral; NYHA: New York Heart Association; OUES: slope da eficiência do consumo de oxigênio; P_ET_CO_2_: pressão parcial de dióxido de carbono expirado; P_ET_CO_2AT_: P_ET_CO_2_ em AT; P_ET_CO_2DIF_: aumento do P_ET_CO_2_ até atingir o AT; pVO_2_: pico de consumo de oxigênio; RER: relação de troca respiratória ; TRC: terapia de ressincronização cardíaca.

### Desfecho primário

**Figura 1:**
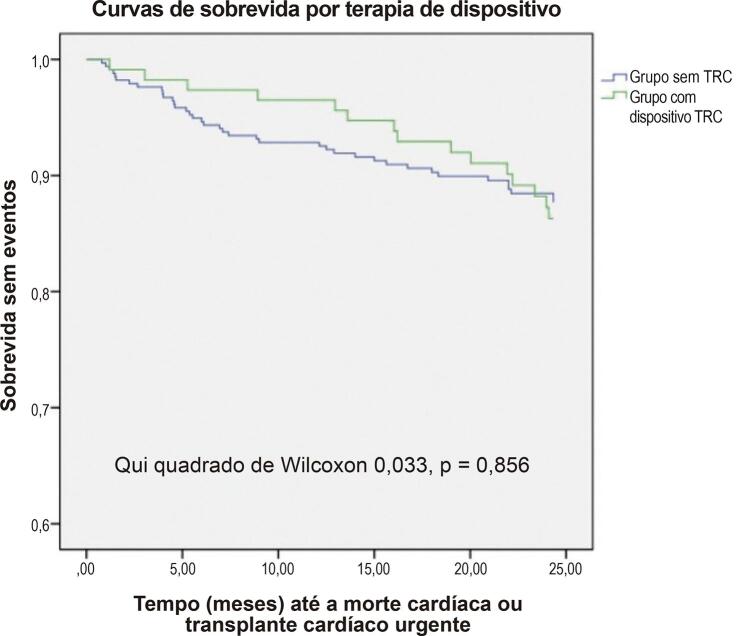
Curvas de sobrevida pela terapia de ressincronização cardíaca.

O desfecho primário ocorreu em 54 (12,0%) pacientes conforme representado na Tabela 2 , sendo 37 pacientes com óbito cardíaco e 16 pacientes com TC de urgência. Uma proporção semelhante de pacientes atingiu o desfecho primário em ambos os grupos, que também se aplicava aos seus componentes individuais. A análise de sobrevida revelou sobrevida livre de eventos semelhante entre os grupos durante o período de acompanhamento ( Figura 1 ).

**Tabela 2:** Eventos adversos em 24 meses de acompanhamento

Eventos adversos em 24 meses de acompanhamento	No geral n (%)	Grupo TRC n (%)	Sem n (%)	valor p
**Desfecho primário combinado**	54 (12,0%)	15 (13,2%)	39 (11,6%)	0,660
Mortalidade total	38 (8,4%)	11 (9,6%)	27 (8,0%)	0,592
Mortalidade cardíaca	37 (8,2%)	11 (9,6%)	26 (7,7%)	0,521
Morte súbita cardíaca	14 (3,1%)	3 (2,6%)	11 (3,3%)	0,977
Morte por piora da IC	23 (5,1%)	8 (7,0%)	15 (4,5%)	0,285
TC urgente	16 (3,6%)	4 (3,5%)	12 (3,6%)	0,991

*TRC: terapia de ressincronização cardíaca; IC: insuficiência cardíaca; TC: transplante cardíaco.*

TRC: terapia de ressincronização cardíaca.

### Relação entre os parâmetros prognósticos do TCPE e o resultado primário

Tanto em pacientes com TRC quanto na coorte total, pVO_2_, slope VE/VCO_2_ e P_ET_CO_2AT_ foram preditores independentes do desfecho primário – Tabela 3 .

**Tabela 3:** TCPE Preditores de eventos adversos em 24 meses de acompanhamento

Coorte Total	Univariado, OR (95% IC)	valor p	Análise multivariada, OR (95% IC)		valor p
pVO_2_ (ml/kg/min)	0,851 (0,799-0,906)	<0,001		0,867 (0,812-0,921)	0,004
Slope VE/VCO_2_	1,092 (1,061-1,124)	0,005		1,104 (1,020-1,196)	0,015
Ponto ótimo cardiorrespiratório	1,128 (1,050-1,212)	0,010			0,250
OUES	0,357 (0,179-0,713)	<0,001			0,284
Potência Circulatória (mmHg.ml.kg-1 min-1)	0,996 (0,994-0,999)	0,040			0,540
Potência ventilatória (mmHg)	0,471(0,367-0,605)	0,017			0,287
Pico de pulso O_2_ (mL/kg/batimento)	0,769 (0,573-1,031)	0,079			0,357
P_ET_CO_2_ em repouso (mmHg)	0,871 (0,814-0,931)	0,012			0,135
P_ET_CO_2AT_ (mmHg)	0,814 (0,763-0,868)	<0,001		0,713 (0,577-0,880)	0,002
P_ET_CO_2DIF_ (mmHg)	0,734 (0,660-0,815)	<0,001			0,110
**Grupo TRC**	**Univariado, OR (95% IC)**	**valor p**		**Análise multivariada, OR (95% IC)**	**valor p**
pVO_2_ (ml/kg/min)	0,794 (0,688-0,916)	0,002		0,821 (0,647-0,905)	0,005
Slope VE/VCO_2_	1,162 (1,077-1,253)	<0,001		1,109 (1,053-1,165)	0,008
Ponto ótimo cardiorrespiratório	1,101 (0,982-1,235)	0,090			0,319
OUES	0,974 (0,702-1,353)	0,470			0,657
Potência Circulatória (mmHg.ml.kg-1 min-1)	0,997 (0,998-0,999)	0,047			0,470
Potência ventilatória (mmHg)	0,313 (0,157-0,624)	0,001			0,314
Pico de pulso O_2_ (mL/kg/batimento)	0,751 (0,371-1,063)	0,097			0,490
P_ET_CO_2_ em repouso (mmHg)	0,779 (0,668-0,910)	0,002			0,197
P_ET_CO_2AT_ (mmHg)	0,564 (0,413-0,771)	<0,001		0,527 (0,309-0,898)	0,001
P_ET_CO_2DIF_ (mmHg)	0,595 (0,451-0,786)	<0,001			0,097
**Grupo Sem TRC**					
pVO_2_ (ml/kg/min)	0,860 (0,801-0,924)	<0,001		0,819 (0,668-0,930)	0,007
Slope VE/VCO_2_	1,075 (1,040-1,110)	<0,001		1,109 (1,015-1,210)	0,012
Ponto ótimo cardiorrespiratório	1,143 (1,040-1,257)	0,005			0,154
OUES	0,088 (0,030-0,253)	<0,001			0,454
Potência Circulatória (mmHg.ml.kg-1 min-1)	0,095 (0,091-0,097)	0,039			0,564
Potência ventilatória (mmHg)	0,513 (0,391-0,674)	<0,001			0,309
Pico de pulso O_2_ (mL/kg/batimento)	0,783 (0,453-1,021)	0,070			0,410
P_ET_CO_2_ em repouso (mmHg)	0,900 (0,834-0,972)	0,007			0,229
P_ET_CO_2AT_ (mmHg)	0,849 (0,794-0,907)	0,001			0,080
P_ET_CO_2DIF_ (mmHg)	0,765 (0,682-0,858)	<0,001		0,689 (0,532-0,893)	0,005

*IC: intervalo de confiança; TCPE: teste cardiopulmonar de exercício; OR: Odds-ratio; NS: não significativo (> 0,05); OUES: slope da eficiência do consumo de oxigênio; P_ET_CO_2_: pressão parcial de dióxido de carbono expirado; P_ET_CO_2AT_: P_ET_CO_2_ no limiar anaeróbico; P_ET_CO_2DIF_: P_ET_CO_2_ aumenta até atingir o limiar anaeróbio; pVO_2_: pico de consumo de oxigênio; TRC: terapia de ressincronização cardíaca.*

No grupo TRC, P_ET_CO_2AT_ apresentou o maior valor de AUC seguido de P_ET_CO_2DIF_ e slope VE/VCO2 – Tabela 4 . O COP apresentou o menor poder preditivo neste grupo. O teste de Hanley & McNeil revelou que o P_ET_CO_2AT_ foi a única variável que apresentou poder preditivo significativamente maior que o do pVO_2_ – Tabela 5 .

**Tabela 4:** Análise de AUC para o desfecho primário

Características	Grupo TRC		Grupo Sem TRC		Hanley e McNeil para comparação da curva ROC entre os grupos (valor p)
	AUC	95% IC	AUC	95% IC	
pVO_2_ (ml/kg/min)	0,778	0,683-0,873	0,723	0,643-0,804	0,531
Slope VE/VCO_2_	0,868	0,782-0,954	0,757	0,693-0,822	0,159
Ponto ótimo cardiorrespiratório	0,668	0,355-0,980	0,739	0,487-0,991	0,699
OUES	0,775	0,591-0,960	0,800	0,710-0,890	0,808
Potência Circulatória (mmHg.ml.kg-1 min-1)	0,777	0,679-0,876	0,743	0,668-0,819	0,697
Potência ventilatória (mmHg)	0,830	0,729-0,930	0,759	0,687-0,830	0,398
Pico de pulso O_2_ (mL/kg/batimento)	0,659	0,486-0,831	0,716	0,642-0,761	0,546
P_ET_CO_2_ em repouso (mmHg)	0,797	0,518-0,713	0,615	0,518-0,713	**0,042**
P_ET_CO_2AT_ (mmHg)	0,951	0,900-0,980	0,741	0,662-0,8220	**0,002**
P_ET_CO_2DIF_ (mmHg)	0,889	0,819-0,960	0,776	0,712-0,841	0,121

*AUC: Área sob a curva; IC: intervalo de confiança; OUES: slope da eficiência do consumo de oxigênio; P_ET_CO_2_: pressão parcial de dióxido de carbono expirado; P_ET_CO_2AT_: P_ET_CO_2_ no limiar anaeróbico; P_ET_CO_2DIF_: P_ET_CO_2_ aumenta até atingir o limiar anaeróbio; pVO_2_: pico de consumo de oxigênio; ROC: curva de operação do receptor; TRC: terapia de ressincronização cardíaca.*

**Tabela 5:** Hanley e McNeil para comparação da curva ROC entre cada variável e pVO2 (valor p)

Características	Grupo TRC	Grupo Sem TRC
Slope VE/VCO_2_	0,353	0,613
Ponto ótimo cardiorrespiratório	0,487	0,900
OUES	0,979	0,261
Potência circulatória (mmHg,ml,kg-1 min-1)	0,992	0,766
Potência ventilatória (mmHg)	0,607	0,592
Pico de pulso O_2_ (mL/kg/batimento)	0,277	0,918
P_ET_CO_2_ em repouso (mmHg)	0,855	0,123
P_ET_CO_2AT_ (mmHg)	**0,046**	0,794
P_ET_CO_2DIF_ (mmHg)	0,213	0,431

*AUC: Área sob a curva; OUES: slope da eficiência do consumo de oxigênio; P_ET_CO_2_: pressão parcial de dióxido de carbono expirado; P_ET_CO_2AT_: P_ET_CO_2_ no limiar anaeróbico; P_ET_CO_2DIF_: P_ET_CO_2_ aumenta até atingir o limiar anaeróbio; pVO_2_: pico de consumo de oxigênio ; TRC: terapia de ressincronização cardíaca.*

No grupo sem TRC, OUES e P_ET_CO_2DIF_ apresentaram os maiores valores de AUC, ambos superiores ao de pVO_2_ e slope VE/VCO2, mas não foi encontrada diferença estatisticamente significativa.

**Figura 2:**
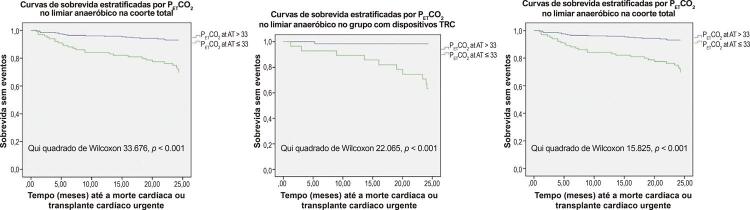
Curvas de sobrevida segundo ponto de corte de PETCO2AT de 33mmHg na coorte geral, grupo TRC e grupo sem TRC ; TRC: terapia de ressincronização cardíaca.

P_ET_CO_2AR_ e P_ET_CO_2AT_ foram os únicos parâmetros que revelaram um melhor desempenho em pacientes com TRC do que em pacientes sem dispositivo – Tabela 4 . Um P_ET_CO_2AT_ de 33mmHg teve sensibilidade de 90% e especificidade de 78% para o desfecho primário no grupo TRC e abaixo desse valor, os pacientes tiveram uma sobrevida de 24 meses livre de eventos significativamente menor, não apenas na coorte total, mas também nos dois grupos de estudo – Figura 2 .

### Valor de corte para seleção de TC

**Figura 3:**
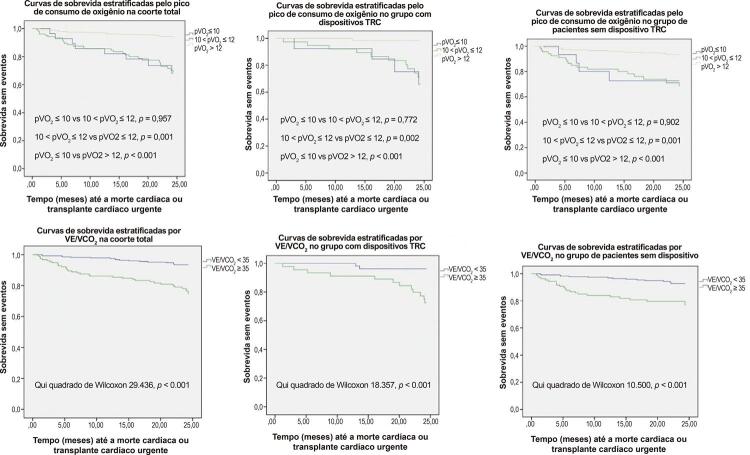
Curvas de sobrevida estratificadas por pVO2 e VE/VCO2 para a coorte total, grupo TRC e grupo sem TRC. TRC: terapia de ressincronização cardíaca.

Na coorte geral, assim como em cada grupo, pacientes com pVO_2_ > 12ml/kg/min (ou > 14ml/kg/min se sob BB)^11^ tiveram melhor prognóstico em comparação com pacientes em estratos com pVO_2_ ≤ 10ml/kg/min e 10 < pVO2 ≤ 12ml/kg/min, enquanto um ponto de corte de 10ml/kg/min não forneceu uma estratificação de risco adequada – Figura 3 . Um ponto de corte do slope VE/VCO2 de 35 discriminou significativamente o risco para eventos de IC em todas as coortes – Figura 3 .

Para o ponto de corte tradicional de pVO_2_ para seleção de TC, VPP para o desfecho primário foi de 98,4% no grupo TRC e 93,3% no grupo sem TRC ( Tabela 5 ), com VPN de 27,5% e 27,2%, respectivamente. Um ponto de corte de pVO_2_ de 10 ml/kg/min revelou VPP menor em ambos os grupos, apesar de ter VPN semelhante, sem diferenças significativas entre os grupos – Tabela 6 .

**Tabela 6:** VPP e VPN de pontos de corte de várias variáveis para o desfecho primário

Características	Grupo TRC		Grupo Sem TRC	
	VPN	VPP	VPN	VPP
pVO_2_ ≤ 10 ml/kg/min	89,0%	30,8%	89,1%	26,7%
pVO_2_ ≤ 12 ml/kg/min^1^	98,4%	27,5%	93,3%	27,2%
VE/VCO_2_ slope ≥ 35	96,4%	26,1%	93,1%	21,7%
P_ET_CO_2AT_ ≤ 33 mmHg	98,4%	35,7%	91,9%	27,4%

*^1^ pVO_2_ ≤ 12 ml/kg/min ou ≤ 14 ml/kg/min sem betabloqueador VPN: valor preditivo negativo; P_ET_CO_2AT_: pressão parcial de dióxido de carbono expirado no limiar anaeróbio; pVO_2_: pico de consumo de oxigênio; VPP: valor preditivo positivo; TRC: terapia de ressincronização cardíaca.*

No grupo TRC, P_ET_CO_2AT_ ≤ 33 mmHg apresentou valores de VPP e VPN ligeiramente maiores do que o ponto de corte de pVO_2_ recomendado.

## Discussão

Ensaios anteriores mostraram que a adição de TRC à terapia médica ideal ou terapia com desfibrilador reduz significativamente a mortalidade entre os pacientes com ICFER^4 , 7^ e melhora a capacidade de exercício, levando a um aumento do pVO_2_ e uma redução do slope VE/VCO2, atrasando com segurança o TC.^18 , 19^ Reconheceu-se a necessidade de revisão dos pontos de corte de seleção de TC devido à melhora nas terapias de IC.^9 , 10^ Com base no benefício de sobrevida conferido pela TRC e seu efeito no pVO_2_, não está claro se esta ainda é uma ferramenta válida para a seleção de TC. Um trabalho de 2011 sugeriu que o HFSS superou o pVO_2_ na estratificação de risco na presença de um CIED e que um ponto de corte de pVO_2_ de 10 ml/kg/min seria mais adequado.^14^ Nossa análise procurou atender a essa necessidade não atendida na cardiologia contemporânea.

Houve diferenças cruciais na linha de base entre os grupos, pois os pacientes do grupo TRC eram significativamente mais velhos, mais sintomáticos, tinham FEVE mais baixa, níveis médios de peptídeos natriuréticos mais altos, maior prevalência de FA e DRC e pior desempenho no exercício – pVO_2_ basal mais baixo e slope VE/VCO2 mais alto. No entanto, isso não se traduziu em pior prognóstico, pois uma proporção semelhante de pacientes atingiu o desfecho primário em ambos os grupos (12,0% vs 13,2%, p = 0,660), sem diferença significativa na sobrevida livre de eventos (p = 0,856).

Como esperado, o pVO_2_ apresentou um poder prognóstico aceitável, independente da presença de um aparelho de TRC (p = 0,531). O slope VE/VCO2 foi sugerido como mais preciso do que os critérios de listagem atuais para TC.^20^ Não houve diferença entre os grupos quanto ao seu poder preditivo (p = 0,159); e quanto à sua capacidade preditiva, apesar de numericamente maior que a do pVO_2_, essa diferença não atingiu significância estatística em nenhum grupo.

A P_ET_CO_2_ correlaciona-se com o débito cardíaco em pacientes com IC e pode refletir a gravidade da doença, tendo valor prognóstico independente do pVO_2_.^21 - 24^ Um P_ET_CO_2AR_ < 33,0 mmHg ou um aumento < 3 mmHg durante o teste ergométrico foram associados a pior prognóstico.^3^ Nos pacientes com TRC, P_ET_CO_2AR_, P_ET_CO_2AT_ e P_ET_CO_2DIF_ apresentaram valores de AUC maiores que pVO_2_, mas essa diferença só atingiu significância estatística para P_ET_CO_2AT_ (p = 0,046). Pacientes com P_ET_CO_2AT_ ≤ 33,0 mmHg tiveram uma sobrevida de 24 meses livre de eventos significativamente menor, não apenas no braço de TRC, mas também na coorte geral e no grupo sem TRC (p < 0,001).

Um valor de corte de pVO_2_ de 10 ml/kg/min não melhorou a estratificação de risco no grupo TRC, uma vez que tem VPN marcadamente menor do que os pontos de corte tradicionais. Não houve discriminação entre os estratos de alto risco (pVO_2_ ≤10 ml/min/kg) e médio risco (10 < pVO2 ≤ 12 ml/min/kg) quanto à sobrevida livre de eventos durante os primeiros 24 meses de seguimento em nenhum dos grupos. Os estratos de baixo risco (pVO_2_ ≥12 ml/min/kg) tiveram prognóstico significativamente melhor do que os demais estratos, em ambos os grupos. O valor de corte recomendado para VE/VCO2 forneceu discriminação de risco precisa de 2 anos no grupo TRC (72,6% vs 96,6%, p = 0,001).

Apesar dos pacientes com TRC apresentarem um perfil basal de risco mais alto em nosso estudo, isso não se traduziu em uma maior taxa de eventos durante o seguimento. O ponto de corte atual de pVO_2_ para seleção de TC pode estratificar esses pacientes de alto risco com mais precisão do que o ponto de corte de pVO_2_ sugerido de 10ml/kg/min,^14^ independentemente da presença de um dispositivo de TRC.

O baixo VPP e o alto VPN das variáveis analisadas sugerem que na população estudada todos esses parâmetros, quando utilizados individualmente, são mais adequados para identificar pacientes que não necessitam de TC.

Nossos resultados sugerem que terapias avançadas de IC podem ser suspensas com segurança em pacientes com IC, com pVO_2_ > 12 ml/kg/min (ou 14 ml/kg/min na ausência de betabloqueador), independentemente da presença de dispositivo de TRC, pois a taxa de eventos nesta população é baixa. Pacientes abaixo desse ponto de corte devem ser tratados de acordo, e seu encaminhamento oportuno para TC ou MCS deve ser considerado. O baixo VPP dos pontos de corte recomendados sugere que o pVO_2_ só é insuficiente para orientar o encaminhamento e outros fatores prognósticos devem ser levados em consideração, como classe funcional NYHA, perfil INTERMACS, FEVE, HFSS, internações recorrentes planejadas e não planejadas por IC ou arritmias ventriculares, congestão persistente/necessidade de doses crescentes de diuréticos ou combinação com outras variáveis do TCPE, como P_ET_CO_2AT_. O VPP surpreendentemente baixo pode ser explicado pelo fato de que uma proporção significativa de nossa coorte realizou um TCPE submáximo, uma configuração na qual o pVO_2_ pode perder poder discriminativo.

O P_ET_CO_2AT_ pode aumentar o valor prognóstico do TCPE em ICFER, independentemente da presença de um dispositivo de TRC, e eventualmente refinar a capacidade preditiva dos parâmetros de TCPE atualmente usados para a decisão de encaminhamento para TC.

### Limitações do estudo

Esta foi uma experiência de centro único e, portanto, os resultados podem refletir nossa prática local e podem não ser aplicáveis a outros Centros de IC.

Em segundo lugar, apesar de um grande número de pacientes estar recebendo terapias de bloqueio neurohormonal aprovadas pelas diretrizes, vários pacientes foram incluídos nesta análise antes do advento dos inibidores do receptor de angiotensina-neprilisina – ARNI (<10% dos pacientes sob ARNI). Portanto, não está claro se nossos resultados podem ser extrapolados para a era sacubitril-valsartana, pois esse medicamento demonstrou ter impacto na capacidade de exercício.

A grande maioria dos pacientes na coorte TRC tinha um dispositivo TRC-D (98,4%), por isso não se sabe se P_ET_CO_2AT_ e outras variáveis TCPE manteriam sua capacidade preditiva em pacientes com dispositivos TRC-P. Como os pacientes do braço da TRC tinham um perfil clínico de base teórica de risco mais alto, seria esperado que, na ausência de um desfibrilador, uma proporção maior desses pacientes atendesse ao desfecho primário, devido a taxas mais altas de morte arrítmica. Quarto, não há dados sobre a resposta de TRC e seria útil comparar o desempenho dessas variáveis entre aqueles que respondem e que não respondem clínica/ecocardiograficamente.

Além disso, o pVO_2_ e outras variáveis do TCPE podem perder parte de seu valor prognóstico em um cenário submáximo.^25^ No entanto, nossa coorte total apresentou um RER médio de 1,07 e o grupo TRC de 1,05, significando que uma proporção substancial de pacientes realizou exercício submáximo, o que pode influenciar no desempenho de cada parâmetro.

## Conclusões

O desempenho das ferramentas de estratificação de risco em pacientes com IC encaminhados para TC foi definido antes da ampla utilização de dispositivos de TRC e há dados limitados sobre sua acurácia prognóstica nesses pacientes. Nossos achados sugerem que os valores de corte recomendados de pVO_2_ e VE/VCO_2_ mantêm sua capacidade discriminativa nesse cenário; no entanto, o P_ET_CO_2AT_ pode fornecer uma maior capacidade preditiva de eventos adversos em um seguimento de 24 meses em pacientes com TRC. Esse parâmetro foi um preditor de prognóstico independente em pacientes com TRC e teve melhor desempenho nessa população do que em pacientes sem TRC. Mais estudos são necessários para avaliar a reprodutibilidade de nossos dados e para avaliar se P_ET_CO_2AT_ pode melhorar a estratificação de risco quando combinado com pVO_2_.
