# Supplementary material for: Capacidade Preditiva dos Parâmetros do Teste de Esforço Cardiopulmonar em Pacientes com Insuficiência Cardíaca em Terapia de Ressincronização Cardíaca
Source: Arq Bras Cardiol. 2022 Jul 13;119(3):413–23. [Article in Portuguese] doi: 10.36660/abc.20210620 (PMC9438531; doi:10.36660/abc.20210620)
Supplement: Translation

# Translation: Predictive Ability of Cardiopulmonary Exercise Test Parameters in Heart Failure Patients with Cardiac Resynchronization Therapy

**Authors:** João Ferreira Reis, António Valentim Gonçalves, Pedro Garcia Brás, Rita Ilhão Moreira, Pedro Rio, Ana Teresa Timóteo, Rui M. Soares, Rui Cruz Ferreira

**Affiliations:** 1 Portugal Departamento de Cardiologia, Hospital de Santa Marta, Centro Hospitalar Central de Lisboa, Lisboa – Portugal

**Keywords:** Heart Failure, Cardiac Resynchronization Therapy/methods, Exercise Test/methods, Oxygen Consumption, Heart Transplantation

## Abstract

**Background:**

There is evidence suggesting that a peak oxygen uptake (pVO_2_) cut-off of 10ml/kg/min provides a more precise risk stratification in cardiac resynchronization therapy (CRT) patients.

**Objective:**

To compare the prognostic power of several cardiopulmonary exercise testing (CPET) parameters in this population and assess the discriminative ability of the guideline-recommended pVO_2_cut-off values.

**Methods:**

Prospective evaluation of consecutive heart failure (HF) patients with left ventricular ejection fraction ≤40%. The primary endpoint was a composite of cardiac death and urgent heart transplantation (HT) in the first 24 follow-up months, and was analysed by several CPET parameters for the highest area under the curve (AUC) in the CRT group. A survival analysis was performed to evaluate the risk stratification provided by several different cut-offs. p values <0.05 were considered significant.

**Results:**

A total of 450 HF patients, of which 114 had a CRT device. These patients had a higher baseline risk profile, but there was no difference regarding the primary outcome (13.2% vs 11.6%, p =0.660). End-tidal carbon dioxide pressure at anaerobic threshold (P_ET_CO_2AT)_had the highest AUC value, which was significantly higher than that of pVO_2_in the CRT group (0.951 vs 0.778, p =0.046). The currently recommended pVO_2_cut-off provided accurate risk stratification in this setting (p <0.001), and the suggested cut-off value of 10 ml/min/kg did not improve risk discrimination in device patients (p =0.772).

**Conclusion:**

P_ET_CO_2AT_may outperform pVO_2_’s prognostic power for adverse events in CRT patients. The current guideline-recommended pVO2 cut-off can precisely risk-stratify this population.

## Introduction

The cardiopulmonary exercise test (CPET) is a powerful predictor of mortality in heart failure patients with reduced ejection fraction (HFrEF) and is used to guide patient referral for advanced therapies, such heart transplantation (HT) and mechanical circulatory support (MCS).^1 - 3^

Peak oxygen uptake (pVO_2_) and the VE/VCO_2_slope are CPET-derived variables most commonly used as risk assessment tools; however, several other CPET variables have been shown to predict HF events and, some of them, can improve clinical stratification of HF patients when used together with the aforementioned variables (i.e., exercise oscillatory ventilation, end-tidal carbon dioxide variation during exercise testing, HR recovery, systolic blood pressure and the ECG response to exercise).

Cardiac resynchronization therapy (CRT) has emerged as a major therapeutic option in the management of HFrEF patients and, in selected patients, has shown to improve symptomatic burden and quality of life, as well to have a prognostic benefit regarding morbidity and mortality.^4 - 8^ A growing number of patients referred to HT already have a CRT device, either with or without defibrillator (CRT-D and CRT-P, respectively). Survival in HFrEF patients has improved significantly in recent years and some authors suggest the need for re-evaluation of the listing criteria for HT and prognostic thresholds of peak oxygen uptake (pVO_2_) and VE/VCO2 slope.^9 , 10^

The *2016 International Society for Heart Lung Transplantation (ISHLT) listing criteria for heart transplantation* defined pVO_2_as a major criterion for listing patients for HT and that the presence of CRT device does not alter the recommended cut-off value of pVO_2._^11^ This recommendation was based on a sub-analysis of the COMPANION trial which showed that CRT did not alter the predictability of pVO_2_on adverse HFrEF events.^12 , 13^ Conversely, Goda et al.^14^ showed that a cut-off value of 10 ml/kg/min rather than the traditional cut-off value of 14 ml/kg/min may be more useful for risk stratification in patients with CRT.^14^ Several other CPET variables were proven to be robust predictors of a worse clinical outcome in HFrEF populations, such as the VE/VCO_2_slope, the O_2_uptake efficiency slope (OUES) and the Cardiorespiratory Optimal Point (COP)^15 , 16^ .

The present study seeks to evaluate the predictive ability of the guideline recommended cut-off values in patients with CRT, to compare the prognostic power of several exercise parameters to that of pVO_2_in this population and to compare their performance between patients with and without a CRT device.

## Methods

### Ethics

The investigation conforms to the principles outlined in the Declaration of Helsinki. The local institutional ethics committee approved the study protocol. All patients provided informed consent.

### Study Sample

Single centre analysis of 450 consecutive HF patients referred to our institution from 2009 to 2018 with left ventricular ejection fraction (LVEF) ≤40% and New York Heart Association (NYHA) class II or III, who were submitted to CPET. All patients were referred for evaluation by the HF team with possible indication for HT or MCS.

### Study Protocol

Patient follow-up included initial evaluation within a period of one month in each patient with: clinical data including aetiology of HF (ischemic vs non-ischemic), implanted cardiac devices (CIED), medication, comorbidities, NYHA class; laboratorial data; electrocardiographic data; echocardiographic data; CPET data; Heart Failure Survival Score (HFSS).

Patients were excluded if one of the following: age <18 years; planned percutaneous coronary revascularization or cardiac surgery; exercise-limiting comorbidities (cerebrovascular disease, musculoskeletal impairment, or severe peripheral vascular disease); previous HT.

Patients who underwent CRT implantation performed CPET and transthoracic echocardiogram at least 6 months after the procedure.

Patients with elective HT during the follow-up period (patients who had indication for HT and a heart become available in the first two year of follow-up) were censured from the analysis at the time of HT.

### Cardiopulmonary exercise testing

A maximal symptom-limited treadmill CPET, defined by peak respiratory exchange rate (RER) >1.05, was performed using the modified Bruce protocol (GE Marquette Series 2000 treadmill). Gas analysis was preceded by calibration of the equipment. Minute ventilation, oxygen uptake and carbon dioxide production were acquired breath-by-breath, using a SensorMedics Vmax 229 gas analyser.

The pVO_2_was defined as the highest 30-second average achieved during exercise and was normalized for body mass. The anaerobic threshold was determined by combining the standard methods (V-slope preferentially and ventilatory equivalents). The VE/VCO_2_slope was calculated by least squares linear regression, using data acquired throughout the whole exercise. COP was measured as the minimum value of the ventilatory equivalent for oxygen (VE/VO_2_minimum). Partial pressure of end-tidal carbon dioxide (P_ET_CO_2_) was reported before exercise (P_ET_CO_2AR_), at anaerobic threshold (P_ET_CO_2AT_) and at peak exercise in mmHg units, and the increase during exercise until the anaerobic threshold is achieved (P_ET_CO_2DIF_) was also calculated. Peak oxygen pulse (PP) was calculated by dividing derived pVO_2_by the maximum heart rate (HR) during exercise and was expressed in millilitres per beat. Circulatory power was calculated as the product of pVO_2_and peak systolic blood pressure and the ventilatory power was calculated by dividing peak systolic blood pressure (BP) by the VE/VCO_2_slope. Several composite parameters of CPET were also automatically calculated.

### Follow-up and endpoint

All patients were followed-up for 24 months from the date of completion of the aforementioned complementary exams.

The primary endpoint was a composite of cardiac death and urgent HT occurring during an unplanned hospitalisation with dependency of inotropes for worsening HF. Data was obtained from the outpatient clinic visits (i.e., both unplanned visits for HF - clinical deterioration requiring iv diuretics - or planned visits for HF medication up-titration, diuretic therapy or routine clinical evaluation by the HF team) and was complemented with a standardised telephone interview to all patients at 24 months of follow-up.

### Statistical analysis

All analyses compare patients with and without a CRT device (CRT and noCRT, respectively). Data was analysed using the software Statistical Package for the Social Science for Windows, version 24.0 (SPSS Inc, Chicago IL).

Baseline characteristics were summarised as frequencies (percentages) for categorical variables, as means and standard deviations for continuous variables when normality was verified and as median and interquartile range when normality was not verified by the Kolmogorov-Smirnov test. The Student’s t-test for independent samples or the Mann-Whitney test (when normality was not confirmed) were used for all comparisons. Chi-Square test or Fisher exact test were used to compare categorical variables.

Multivariate analysis for the prediction of the primary endpoint during two-years follow-up was performed using Cox regression, by including all statistically significant variables in the univariate analysis, in the total cohort and in each group.

The predictive power of several CPET parameters regarding the primary outcome in each group was analysed with Receiver Operating Characteristics (ROC) curve and area under the curve (AUC). Cut-off values for variables were determined from ROC curves so that the sum of sensitivity and specificity was maximised. Hanley and McNeil test was used to compare two correlated ROC curves.^17^

Event-free survival was determined using the Kaplan- Meier method and compared with log-rank analysis in order to evaluate the risk discriminative ability provided by the guideline-recommended cut-off values of pVO_2_(pVO_2_≤ 12 ml/kg/min or ≤ 14 ml/kg/min without beta-blocker - BB) and VE/VCO_2_slope^11^ and the suggested cut-off value of 10ml/kg/min.^14^ Statistical differences with a p value <0.05 were considered significant.

## Results

### Overview of CRT and noCRT groups

A total of 450 patients were enrolled in the study, of which 25.3% (n = 114) had a CRT device, mostly a CRT-D (98.2%). The overall population had a mean age of 56.2 years, with 78.7% being male and a mean LVEF of 28.6%. All CRT patients with atrial fibrillation underwent AV node ablation during the implantation procedure and the percentage of biventricular pacing was 96%. CPET was performed on average 8 months after CRT implantation. The baseline characteristics of both groups are presented in Table 1 .

**Table 1:** Baseline Characteristics of CRT and no CRT groups

			Overall n 450	CRT n 114	no CRT n 336	p value
			CLINICAL DATA – CHARACTERISTICS			
Age			56.2 ± 12.5	62.3 ± 11.5	54.2 ± 12.2	< 0.001
Male (%)			354 (78.7%)	85 (74.6%)	269 (80.1%)	0.216
BMI (kg/m_2_)			27.2 ± 4.3	27.2 ± 4.1	27.1 ± 4.4	0.829
Ischemic aetiology (%)			211 (46.9%)	42 (36.8%)	169 (50.6%)	0.011
ACEi/ARB/ARNI (%)			423 (94.0%)	104 (96.3%)	319 (96.1%)	1.000
BB (%)			388 (86.2%)	93 (85.3%)	295 (88.9%)	0.325
MRA (%)			340 (75.6%)	93 (84.5%)	247 (74.2%)	0.026
Diabetes (%)			98 (21.8%)	23 (22.3%)	75 (23.4%)	0.817
**CKD (%)**			140 (31.1%)	48 (46.6%)	92 (32.1%)	**0.008**
**AF (%)**			112 (24.9%)	43 (38.1%)	69 (20.6%)	**< 0.001**
**ICD (%)**			271 (60.2%)	112 (98.2%)	159 (47.3%)	**< 0.001**
**NYHA Functional Class**			2.2 ± 0.6	2.5 ± 0.5	2.1 ± 0.6	**0.001**
HFSS*			8.5 ± 1.0	8.14 ± 0.86	8.65 ± 1.04	**< 0.001**
			**LABORATORIAL DATA**			
**Creatinine (mg/dL)**			1.4 ± 0.7	1.6 ± 0.4	1.0 ± 0.3	**0.041**
Sodium (mEq/L)			137.9 ± 3.1	137.5 ± 3.4	138.5 ± 2.9	0.138
**NT-proBNP (pg/ml)**			2224.2 ± 2764.0	2769.7 ± 2575.4	2034.3 ± 2808.1	**0.045**
			**ECHOCARDIOGRAPHIC DATA**			
**LVEDD (mm/m** ^**2**^ )*			35.5 ± 5.9	37.9 ± 5.5	34.7 ± 5.9	**0.032**
**LVEF (%)**			28.6 ± 6.9	26.2 ± 7.2	29.6 ± 6.6	**< 0.001**
MR III-IV (%)			65 (14.7%)	16 (14.0%)	49 (14.5%)	0.935
			**CPET DATA**			
**CPET duration (min)**			9.6 ± 4.4	7.4 ± 4.1	10.3 ± 4.3	**< 0.001**
Peak RER			1.07 ± 0.11	1.05 ± 0.11	1.08 ± 0.10	0.139
**pVO**_**2**_ **(ml/kg/min)**			17.9 ± 6.1	15.2 ± 5.1	18.8 ± 6.1	**< 0.001**
**VE/VCO**_**2**_ **slope**			33.8 ± 9.5	35.8 ± 10.9	33.2 ± 8.9	**0.026**
OUES			2.1 ± 1.8	2.2 ± 2.2	2.0 ± 1.6	0.645
**pVO**_**2**_ **(ml/kg/min) at AT**			13.1 ± 4.5	10.3 ± 3.4	13.8 ± 4.5	**0.001**
**O**_**2**_ **Pulse (mL/kg/beat)**			0.14 ± 0.06	0.12 ± 0.04	0.14 ± 0.07	**0.028**
**Circulatory Power (mmHg.ml.kg-1 min-1)**			2786.9 ± 1578.8	2262.3 ± 965.4	2963 ± 1702.4	**< 0.001**
**Ventilatory Power (mmHg)**			4.8 ± 1.7	4.4 ± 1.7	4.9 ± 1.7	**0.020**
Cardiorespiratory Optimal Point			29.6 ± 7.4	30.7 ± 7.5	29.3 ± 7.4	0.274
P_ET_CO_2_at rest (mmHg)			33.4 ± 4.7	32.9 ± 4.8	33.6 ± 4.7	0.241
**P**_**ET**_ **CO**_**2AT**_ **(mmHg)**			36.7 ± 5.9	35.3 ± 5.9	37.1 ± 5.9	**0.010**
**P**_**ET**_ **CO**_**2DIF**_ **(mmHg)**			3.3 ± 3.7	2.3 ± 3.2	3.6 ± 3.8	**0.004**

Values are mean ± standard deviation or median (interquartile range);p values are calculated by Student´s T-test for independent samples or Mann-Whitney U test as appropriate; chi square test or Fisher exact test were used to compare categorical variables. *Variables with normal distribution. AT: anaerobic threshold; ACEi: angiotensin-converting enzyme inhibitors; ARB: angiotensin receptor blockers; ARNI: angiotensin receptor-neprilysin inhibitors; AF: Atrial fibrillation; BB: Beta-blockers; BMI: body mass index; CPET: cardiopulmonary exercise test; CKD: chronic kidney disease; HFSS: Heart Failure Survival Score; ICD: implantable cardioverter defibrillator; LVEF: left ventricular ejection fraction; LVEDD: left ventricular end-diastolic diameter; MRA: mineralocorticoid receptor antagonists; MR: Mitral regurgitation; NYHA: New York Heart Association; OUES: oxygen uptake efficiency slope; P_ET_CO_2_: partial pressure of end-tidal carbon dioxide; P_ET_CO_2AT_: P_ET_CO_2_at AT; P_ET_CO_2DIF_: P_ET_CO_2_increase until the AT is achieved; pVO_2_: peak oxygen uptake; RER: respiratory exchange ratio; CRT: cardiac resynchronization therapy.

### Primary endpoint

The primary endpoint occurred in 54 (12.0%) patients as represented in Table 2 , with 37 patients experiencing cardiac death and 16 patients undergoing urgent HT. A similar proportion of patients met the primary endpoint in both groups, which also applied to its individual components. Survival analysis revealed a similar event-free survival between groups during the follow-up period ( Figure 1 ).

**Table 2:** Adverse events at 24 months follow-up

Adverse events at 24 months follow-up	Overal n (%)	CRT Group n (%)	No CRT n (%)	p value
**Combined primary endpoint**	54 (12.0%)	15 (13.2%)	39 (11.6%)	0.660
Total mortality	38 (8.4%)	11 (9.6%)	27 (8.0%)	0.592
Cardiac mortality	37 (8.2%)	11 (9.6%)	26 (7.7%)	0.521
Sudden cardiac death	14 (3.1%)	3 (2.6%)	11 (3.3%)	0.977
Death for worsening HF	23 (5.1%)	8 (7.0%)	15 (4.5%)	0.285
Urgent HT	16 (3.6%)	4 (3.5%)	12 (3.6%)	0.991

*CRT: cardiac resynchronization therapy; HF: heart failure; HT: heart transplantation.*

**Figure 1:** Survival curves by cardiac resynchronization therapy. CRT: cardiac resynchronization therapy.

### Relationship between CPET prognostic parameters and primary outcome

Both in patients with CRT and in the total cohort, pVO_2_, VE/VCO_2_slope and P_ET_CO_2AT_were independent predictors of the primary endpoint – Table 3 .

**Table 3:** CPET Predictors of adverse events at 24 months follow-up

Total Cohort	Univariate, OR (CI 95%)	p value	Multivariate analysis, OR (CI 95%)		p value
pVO_2_(ml/kg/min)	0.851 (0.799-0.906)	<0.001		0.867 (0.812-0.921)	0.004
VE/VCO_2_slope	1.092 (1.061-1.124)	0.005		1.104 (1.020-1.196)	0.015
Cardiorespiratory Optimal Point	1.128 (1.050-1.212)	0.010			0.250
OUES	0.357 (0.179-0.713)	<0.001			0.284
Circulatory Power (mmHg.ml.kg-1 min-1)	0.996 (0.994-0.999)	0.040			0.540
Ventilatory Power (mmHg)	0.471(0.367-0.605)	0.017			0.287
Peak O_2_Pulse (mL/kg/beat)	0.769 (0.573-1.031)	0.079			0.357
P_ET_CO_2_at rest (mmHg)	0.871 (0.814-0.931)	0.012			0.135
P_ET_CO_2AT_(mmHg)	0.814 (0.763-0.868)	<0.001		0.713 (0.577-0.880)	0.002
P_ET_CO_2DIF_(mmHg)	0.734 (0.660-0.815)	<0.001			0.110
**CRT Group**	**Univariate, OR (CI 95%)**	**p value**		**Multivariate analysis, OR (CI 95%)**	**p value**
pVO_2_(ml/kg/min)	0.794 (0.688-0.916)	0.002		0.821 (0.647-0.905)	0.005
VE/VCO_2_slope	1.162 (1.077-1.253)	<0.001		1.109 (1.053-1.165)	0.008
Cardiorespiratory Optimal Point	1.101 (0.982-1.235)	0.090			0.319
OUES	0.974 (0.702-1.353)	0.470			0.657
Circulatory Power (mmHg.ml.kg-1 min-1)	0.997 (0.998-0.999)	0.047			0.470
Ventilatory Power (mmHg)	0.313 (0.157-0.624)	0.001			0.314
Peak O_2_Pulse (mL/kg/beat)	0.751 (0.371-1.063)	0.097			0.490
P_ET_CO_2_at rest (mmHg)	0.779 (0.668-0.910)	0.002			0.197
P_ET_CO_2AT_(mmHg)	0.564 (0.413-0.771)	<0.001		0.527 (0.309-0.898)	0.001
P_ET_CO_2DIF_(mmHg)	0.595 (0.451-0.786)	<0.001			0.097
**No CRT Group**					
pVO_2_(ml/kg/min)	0.860 (0.801-0.924)	<0.001		0.819 (0.668-0.930)	0.007
VE/VCO_2_slope	1.075 (1.040-1.110)	<0.001		1.109 (1.015-1.210)	0.012
Cardiorespiratory Optimal Point	1.143 (1.040-1.257)	0.005			0.154
OUES	0.088 (0.030-0.253)	<0.001			0.454
Circulatory Power (mmHg.ml.kg-1 min-1)	0.095 (0.091-0.097)	0.039			0.564
Ventilatory Power (mmHg)	0.513 (0.391-0.674)	<0.001			0.309
Peak O_2_Pulse (mL/kg/beat)	0.783 (0.453-1.021)	0.070			0.410
P_ET_CO_2_at rest (mmHg)	0.900 (0.834-0.972)	0.007			0.229
P_ET_CO_2AT_(mmHg)	0.849 (0.794-0.907)	0.001			0.080
P_ET_CO_2DIF_(mmHg)	0.765 (0.682-0.858)	<0.001		0.689 (0.532-0.893)	0.005

*CI: confidence interval; CPET: cardiopulmonary exercise test; OR: Odds-ratio; NS: not significant (> 0.05); OUES: oxygen uptake efficiency slope; P_ET_CO_2_: partial pressure of end-tidal carbon dioxide; P_ET_CO_2AT_: P_ET_CO_2_at anaerobic threshold; P_ET_CO_2DIF_: P_ET_CO_2_increase until the anaerobic threshold is achieved; pVO_2_: peak oxygen uptake. CRT: cardiac resynchronization therapy.*

In the CRT group, P_ET_CO_2AT_had the highest AUC value followed by P_ET_CO_2DIF_and VE/VCO_2_slope – Table 4 . COP presented the lowest predictive power in this group. The Hanley & McNeil test revealed that P_ET_CO_2AT_was the only variable presenting a significantly higher predictive power than that of pVO_2_– Table 5 .

**Table 4:** AUC analysis for the Primary Endpoint

Characteristics	CRT Group		No CRT Group		Hanley and McNeil for ROC curve comparison between groups (p value)
	AUC	CI 95%	AUC	CI 95%	
pVO_2_(ml/kg/min)	0.778	0.683-0.873	0.723	0.643-0.804	0.531
VE/VCO_2_slope	0.868	0.782-0.954	0.757	0.693-0.822	0.159
Cardiorespiratory Optimal Point	0.668	0.355-0.980	0.739	0.487-0.991	0.699
OUES	0.775	0.591-0.960	0.800	0.710-0.890	0.808
Circulatory Power (mmHg.ml.kg-1 min-1)	0.777	0.679-0.876	0.743	0.668-0.819	0.697
Ventilatory Power (mmHg)	0.830	0.729-0.930	0.759	0.687-0.830	0.398
Peak O_2_Pulse (mL/kg/beat)	0.659	0.486-0.831	0.716	0.642-0.761	0.546
P_ET_CO_2_at rest (mmHg)	0.797	0.518-0.713	0.615	0.518-0.713	**0.042**
P_ET_CO_2AT_(mmHg)	0.951	0.900-0.980	0.741	0.662-0.8220	**0.002**
P_ET_CO_2DIF_(mmHg)	0.889	0.819-0.960	0.776	0.712-0.841	0.121

*AUC: Area under the curve; CI: confidence interval; OUES: oxygen uptake efficiency slope; P_ET_CO_2_: partial pressure of end-tidal carbon dioxide; P_ET_CO_2AT_: P_ET_CO_2_at anaerobic threshold; P_ET_CO_2DIF_: P_ET_CO_2_increase until the anaerobic threshold is achieved; pVO_2_: peak oxygen uptake; ROC: receiver operating curve. CRT: cardiac resynchronization therapy.*

**Table 5:** Hanley and McNeil for ROC curve comparison between each variable and pVO2 (p value

Characteristics	CRT Group	No CRT Group
VE/VCO_2_slope	0.353	0.613
Cardiorespiratory Optimal Point	0.487	0.900
OUES	0.979	0.261
Circulatory Power (mmHg.ml.kg-1 min-1)	0.992	0.766
Ventilatory Power (mmHg)	0.607	0.592
Peak O_2_Pulse (mL/kg/beat)	0.277	0.918
P_ET_CO_2_at rest (mmHg)	0.855	0.123
P_ET_CO_2AT_(mmHg)	**0.046**	0.794
P_ET_CO_2DIF_(mmHg)	0.213	0.431

*AUC: Area under the curve; OUES: oxygen uptake efficiency slope; P_ET_CO_2_: partial pressure of end-tidal carbon dioxide; P_ET_CO_2AT_: P_ET_CO_2_at anaerobic threshold; P_ET_CO_2DIF_: P_ET_CO_2_increase until the anaerobic threshold is achieved; pVO_2_: peak oxygen uptake; CRT: cardiac resynchronization therapy.*

In the noCRT group, OUES and P_ET_CO_2DIF_presented the highest AUC values, both higher than the one of pVO_2_and VE/VCO_2_slope, but no statistically significant difference was found.

P_ET_CO_2AR_and P_ET_CO_2AT_were the only parameters revealing a better performance in patients with CRT than in patients without device – Table 4 . A P_ET_CO_2AT_of 33mmHg had a sensitivity of 90% and a specificity of 78% for the primary outcome in the CRT group and below this value, patients had a significantly lower 24 months survival free of events, not only in the total cohort, but also in the two study groups – Figure 2 .

**Figure 2:** Survival curves according to a PETCO2AT cut-off of 33mmHg in the general cohort, CRT group and no CRT group.

### Cut-off value for HT selection

In the overall cohort, as well as in each group, patients with a pVO_2_> 12ml/kg/min (or > 14ml/kg/min if under BB)^11^ had a better prognosis in comparison to pVO_2_≤ 10ml/kg/min and 10 < pVO_2_≤ 12ml/kg/min strata, whereas a cut-off of 10ml/kg/min did not provide a proper risk stratification – Figure 3 . A VE/VCO_2_slope cut-off of 35 significantly discriminated the risk for HF events in all cohorts – Figure 3 .

**Figure 3:** Survival curves stratified by pVO2 and VE/VCO2 for the total cohort, CRT group and no CRT group. CRT: cardiac resynchronization therapy.

For the traditional pVO_2_cut-off for HT selection, the PPV for the primary outcome was 98.4% in the CRT group and 93.3% in the no CRT group ( Table 5 ), with a NPV of 27.5% and 27.2%, respectively. A pVO_2_cut-off of 10 ml/kg/min revealed a lower PPV in both groups, despite a similar NPV, with no significant differences between groups – Table 6 .

**Table 6:** PPV and NPV of several variables’ cut-offs for the primary endpoint

Characteristics	CRT Group		No CRT Group	
	NPV	PPV	NPV	PPV
pVO_2_≤ 10 ml/kg/min	89.0%	30.8%	89.1%	26.7%
pVO_2_≤ 12 ml/kg/min^1^	98.4%	27.5%	93.3%	27.2%
VE/VCO_2_slope ≥ 35	96.4%	26.1%	93.1%	21.7%
P_ET_CO_2AT_≤ 33 mmHg	98.4%	35.7%	91.9%	27.4%

*^1^pVO_2_≤ 12 ml/kg/min ou ≤ 14 ml/kg/min without beta-blocker NPV: Negative predictive value; P_ET_CO_2AT_: partial pressure of end-tidal carbon dioxide at anaerobic threshold; pVO_2_: peak oxygen uptake; PPV: Positive predictive value; CRT: cardiac resynchronization therapy.*

In the CRT group, P_ET_CO_2AT_≤ 33 mmHg had slightly higher PPV and NPV values than the recommended pVO_2_cut-off.

## Discussion

Previous trial have shown that the addition of CRT to optimal medical therapy or defibrillator therapy significantly reduces mortality among patients with HFrEF^4 , 7^ and improves exercise capacity, leading to an increase in pVO_2_and a reduction of VE/VCO_2_slope, thereby safely delaying HT.^18 , 19^ It has been recognized the need to review HT selection cut-offs due to the improvement in HF therapies.^9 , 10^ Based on the survival benefit conferred by CRT, and its effect on pVO_2_, it is unclear whether this is still a valid tool for HT selection. A work from 2011 suggested that the HFSS outperformed pVO_2_in risk stratification in the presence of a CIED and that a pVO_2_cut-off of 10 ml/kg/min would be more suitable.^14^ Our analysis tried to address this unmet need in contemporary cardiology.

There were crucial baseline differences between groups, as patients in CRT group were significantly older, more symptomatic, had a lower LVEF, higher mean natriuretic peptides levels, higher prevalence of AF and CKD, and a poorer exercise performance – lower baseline pVO_2_and higher VE/VCO_2_slope. However, this did not translate into a worse prognosis, as a similar proportion of patients met the primary endpoint in both groups (12.0% vs 13.2%, p = 0.660), with no significant difference in event-free survival (p = 0.856).

As expected, pVO_2_presented an acceptable prognostic power, irrespective of the presence of a CRT device (p = 0.531). The VE/VCO_2_slope has been suggested to be more accurate than the current listing criteria for HT.^20^ There was no difference between groups regarding its predictive power (p = 0.159), and its predictive ability, despite being numerically higher than that of pVO_2_, this difference did not reach statistical significance in any group.

P_ET_CO_2_correlates with cardiac output in HF patients and can reflect disease severity, having a prognostic value independent of that of pVO_2._^21 - 24^ A P_ET_CO_2AR_< 33.0 mmHg or an increase < 3 mmHg during exercise test were associated with a worse prognosis.^3^ In CRT patients, P_ET_CO_2AR_, P_ET_CO_2AT_and P_ET_CO_2DIF_presented higher AUC values than pVO_2_, but this difference only reached statistical significance for P_ET_CO_2AT_(p = 0.046). Patients with a P_ET_CO_2AT_≤ 33.0 mmHg had a significantly lower 24-months survival free of events, not only in the CRT arm, but also in the overall cohort and in the no CRT group (p < 0.001).

A pVO_2_cut-off value of 10 ml/kg/min did not improve risk stratification in the CRT group, since it has a markedly lower NPV than the traditional cut-offs. There was no discrimination between the high-risk (pVO_2_≤10 ml/min/kg) and the medium risk strata (10 < pVO_2_≤ 12 ml/min/kg) regarding event-free survival during the first 24 months of follow-up in neither of the groups. The low-risk strata (pVO_2_≥12 ml/min/kg) had a significantly better prognosis than the remainder strata, in both groups. The recommended cut-off value for VE/VCO_2_provided accurate 2 years-risk discrimination in the CRT group (72.6% vs 96.6%, p = 0.001).

Despite CRT patients having a higher risk baseline profile in our study, this did not translate into a higher rate of events during follow-up. The current cut-off of pVO_2_for HT selection can stratify these high-risk patients more precisely than the suggested pVO_2_cut-off of 10ml/kg/min,^14^ irrespective of the presence of a CRT device.

The low PPV and the high NPV of the analysed variables suggest that in the studied population all these parameters, when used individually, are best suited to identify patients who do not need HT.

Our results suggest that advanced HF therapies can be safely withheld in HF patients, with pVO_2_> 12 ml/kg/min (or 14 ml/kg/min in the absence of beta-blocker), irrespective of the presence of CRT device, as the event-rate in these population is low. Patients below this cut-off should be managed accordingly, and their timely referral for HT or MCS should be considered. The low PPV of the recommended cut-offs suggests that pVO_2_alone is insufficient to guide referral and other prognostic factors must be taken into account, such as, NYHA functional class, INTERMACS profile, LVEF, HFSS, recurrent planned and unplanned hospitalizations for HF or ventricular arrhythmias, persistent congestion/need for escalating diuretic doses or combining it with other CPET variables, such as P_ET_CO_2AT_. The surprisingly low PPV might be explained by the fact that a significant proportion of our cohort performed a submaximal CPET, a setting on which pVO_2_may lose discriminative power.

P_ET_CO_2AT_may increase the prognostic value of CPET in HFrEF, irrespective of the presence of a CRT device, and eventually refine the predictive ability of the current CPET parameters used for HT referral decision.

### Study limitations

This was a single-centre experience, and therefore, the results can reflect our local practice and might not be applicable to other HF Centres.

Secondly, despite a high number of patients were receiving guideline-approved neurohormonal blockade therapies, several patients were included in this analysis before the advent of angiotensin receptor-neprilysin inhibitors – ARNI (<10% of patients under ARNI). So, it is unclear if our results can be extrapolated to the sacubitril-valsartan era, as this drug has shown to have an impact on exercise capacity.The vast majority of the patients in the CRT cohort had a CRT-D device (98.4%), so it is unknown whether P_ET_CO_2AT_and other CPET variables would retain their predictive ability in patients with

CRT-P devices. As patients in the CRT arm had a theoretical higher risk baseline clinical profile, it would be expected that in the absence of a defibrillator, a higher proportion of these patients would meet the primary endpoint, due to higher rates of arrhythmic death. Fourth, there are no data regarding CRT response and it would be useful to compare these variables’ performance between clinical/ echocardiographic responder and non-responders.Furthermore, pVO_2_and other CPET variables may lose some of their prognostic value in a submaximal setting.^25^ However, our total cohort presented a mean RER of 1.07 and the CRT group of 1.05, meaning that a substantial proportion of patients performed submaximal exercise, which may have an influence on each parameter’s performance.

## Conclusions

The performance of risk stratification tools in HF patients referred for HT was defined before the widespread use of CRT devices and there is limited data regarding their prognostic accuracy in these patients. Our findings suggest that the recommended pVO_2_and VE/VCO_2_cut-off values retain their discriminative ability in this setting; however, P_ET_CO_2AT_may provide a higher predictive ability for adverse events in a 24-months follow-up in CRT patients. This parameter was an independent prognostic predictor in CRT patients and had a better performance in this population than in patients without a CRT. Further studies are required to assess the reproducibility of our data and if P_ET_CO_2AT_can improve risk stratification when combined with pVO_2_.
